# Inflammation: the link between comorbidities, genetics, and Alzheimer’s disease

**DOI:** 10.1186/s12974-018-1313-3

**Published:** 2018-09-24

**Authors:** Estella A. Newcombe, Judith Camats-Perna, Mallone L. Silva, Nicholas Valmas, Tee Jong Huat, Rodrigo Medeiros

**Affiliations:** 1 Neurula Laboratory, Clem Jones Centre for Ageing Dementia Research, Queensland Brain Institute, The University of Queensland, Building 79, Brisbane, 4072 QLD Australia; 2 Queensland Brain Institute, The University of Queensland, Brisbane, 4072 QLD Australia; 3 Centre for Stem Cell Ageing and Regenerative Engineering, The University of Queensland, Brisbane, 4072 QLD Australia

**Keywords:** Alzheimer’s disease, Microglia, Aging, Neuroinflammation, APOE4, TREM2, CD33, Diabetes, TBI, Obesity

## Abstract

Alzheimer’s disease (AD) is a neurodegenerative disorder, most cases of which lack a clear causative event. This has made the disease difficult to characterize and, thus, diagnose. Although some cases are genetically linked, there are many diseases and lifestyle factors that can lead to an increased risk of developing AD, including traumatic brain injury, diabetes, hypertension, obesity, and other metabolic syndromes, in addition to aging. Identifying common factors and trends between these conditions could enhance our understanding of AD and lead to the development of more effective treatments. Although the immune system is one of the body’s key defense mechanisms, chronic inflammation has been increasingly linked with several age-related diseases. Moreover, it is now well accepted that chronic inflammation has an important role in the onset and progression of AD. In this review, the different inflammatory signals associated with AD and its risk factors will be outlined to demonstrate how chronic inflammation may be influencing individual susceptibility to AD. Our goal is to bring attention to potential shared signals presented by the immune system during different conditions that could lead to the development of successful treatments.

## Background

**Fig. 1:**
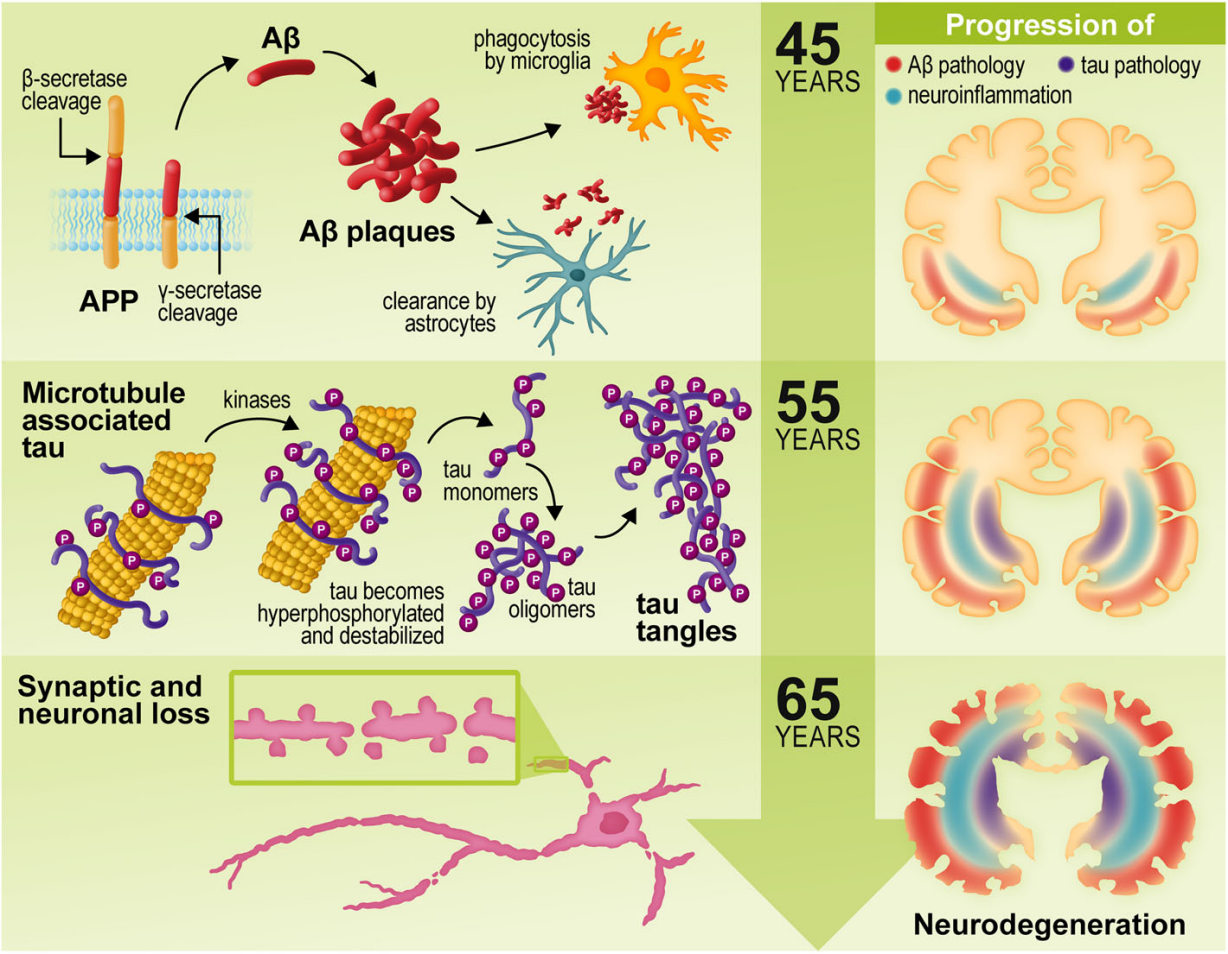
The pathogenic hallmarks of AD in the human brain over time. In the earliest stages of AD, the formation of Aβ occurs due to abnormal cleavage of amyloid precursor protein (APP) by β- and γ-secretases, whereas it is normally cleaved by α- and γ-secretases. Aβ monomers are intrinsically disordered and have a propensity to oligomerize and aggregate into Aβ plaques. Aβ activates microglia and astrocytes, causing them to clear Aβ via phagocytosis and proteolysis. The presence of Aβ has also been linked to the hyperphosphorylation and destabilization of tau and the subsequent formation of tau tangles. Inflammatory activation and signaling can also cause further production of Aβ. Tau pathology is observed approximately 10 years after the initiation of Aβ aggregation. Tau is a microtubule-associated protein that is predominately found in neurons, where it is regulated by phosphorylation and other post-translational modifications (i.e., acetylation, ubiquitylation) to stabilize microtubules, regulate axonal stability, and maintain cell function. While tau contains 2–3 mol of phosphate in the healthy brain, it can accumulate up to three times more phosphate in AD. This hyperphosphorylation lowers the affinity of tau for the microtubules, increases its resistance to degradation by proteases and the proteasome, and leads to its fibrillization and aggregation into neurofibrillary tangles, which ultimately causes neuronal loss and cognitive decline. The increase in Aβ aggregation, inflammation, and tau hyperphosphorylation leads to a variety of downstream effects on neuronal synapses, including inhibition of LTP, impaired dendritic trafficking, increased excitotoxicity, and a reduction in synaptic density. This leads to synaptic loss and eventual neuronal loss approximately 20 years following initial disease pathogenesis, which is followed by the symptomatic cognitive decline

Alzheimer’s disease (AD), classified as a type of dementia, is a neurodegenerative disease which has come into the spotlight due to its high prevalence in the elderly. Currently, the disease impacts 1 in 10 people over 65 years of age and 1 in 3 people over 85. Initial symptoms include memory loss, cognitive impairment, and confusion, progressing to full debilitation over time. The neuropathological processes associated with the disease, including the accumulation of amyloid plaques and neurofibrillary tangles (NFTs), inflammation, and synaptic and neuronal loss, are predicted to build up for 10–20 years before the clinical symptoms arise, representing the largest obstacle in the development of effective treatments for the disease (Fig. 1).

Currently, two classes of drugs—cholinesterase inhibitors (i.e., donepezil, galantamine and rivastigmine) and *N*-methyl-d-aspartate receptor (NMDAR) antagonists (i.e., memantine)—are approved to treat AD; however, the efficacy of these drugs leaves a lot to be desired. No new drug has been approved since Namenda (memantine) in 2003, despite the extensive number of clinical trials aimed at tackling the disease [1]. Although the goal is to cure AD and reverse its harmful effects, any efficacious strategy to slow or prevent the progression of the disease would have a tremendous global socio-economic impact in the current epidemiological scenario. To design such a strategy, however, we require a better understanding of how AD starts and what makes some individuals resilient to the disease. The hope is that this knowledge will lead to the identification of cellular and molecular events that could be targeted therapeutically.

In this review, we highlight how risk and protective factors that are associated with AD affect the immune system. We also discuss the evidence indicating that the long-lasting dysregulation of inflammatory responses impact brain function, thereby facilitating the onset and progression of neurodegeneration in AD. Although we provide some discussion of how the immune system responds to AD, our major focus is to discuss how it triggers important features of the disease, namely β-amyloid (Aβ) accumulation, tau pathology, synaptic and neuronal loss, and cognitive impairment. We also highlight potential shared signals presented by the immune system during different conditions in the body and brain that could lead to the development of strategies for the management of the disease.

## The foundations of neuroinflammation

**Fig. 2:**
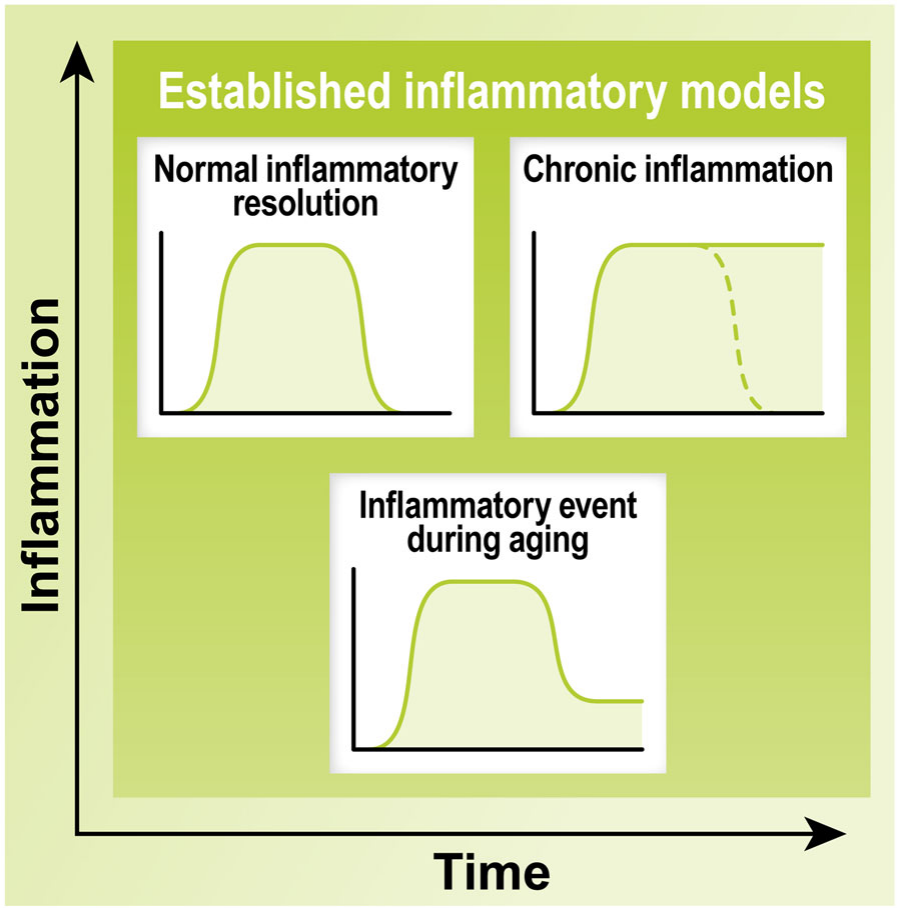
Time-course of pro- and anti-inflammatory responses. The accepted model of acute inflammation shows the activation and resolution of inflammation that occurs due to pro- and then anti-inflammatory modulators. These processes become dysfunctional during chronic inflammation where the resolution phase is not achieved due to excessive pro-inflammatory signaling. Considering more recent developments in the studies of inflammation, we propose a hypothetical model of inflammation during aging, in which an event is not resolved to a baseline inflammatory level, but leaves some residual pro-inflammatory effects (i.e., microglial priming)

Inflammation is a vital physiological immune response against a myriad of factors including infection, trauma, and disease. Not surprisingly, its failure provokes substantial detrimental effects. Contrasting examples of immune dysregulation are the deficiency of the immune response caused by the human immunodeficiency virus and the chronic immune activation in the autoimmune disease multiple sclerosis. In a proper response, immune cells are recruited to the area where the insult occurred via pro-inflammatory signaling pathways. Once recruited, these cells can initiate many different activities, such as increasing vascularization, recruiting additional immune cells via pro-inflammatory signaling, and initiating the phagocytosis of debris and pathogens. The mediators involved in the onset of systemic immune responses are pro-inflammatory and include transcriptional factors (e.g., NF-κB), peptides (e.g., bradykinin), cytokines (e.g., IL-1β, IL-6, IL-18, TNF-α, IFN-γ), chemokines (e.g., CCL2, CCL3, CXCL8), complement (e.g., C1q, C5), enzymes (e.g., COX-2, iNOS, LOX), lipids (e.g., PGE_2_), and coagulation factors (e.g., platelet activating factor). When the trigger of the response is successfully neutralized, immune cells shift their activity towards a pro-resolution phenotype via anti-inflammatory signaling, including lipoxins (e.g., LXA_4_, RvE1) and cytokines (e.g., IL-10, IL-37, TGF-β). The resolution of inflammation involves the downregulation of pro-inflammatory mediators and the increased expression of anti-inflammatory mediators, with its major purposes being to promote the clearance of debris and the repair of the injured tissue [2]. Acute inflammatory events are resolved relatively quickly, with levels of inflammation returning to baseline; however, resolution is not achieved in cases of chronic inflammation (Fig. 2).

**Fig. 3:**
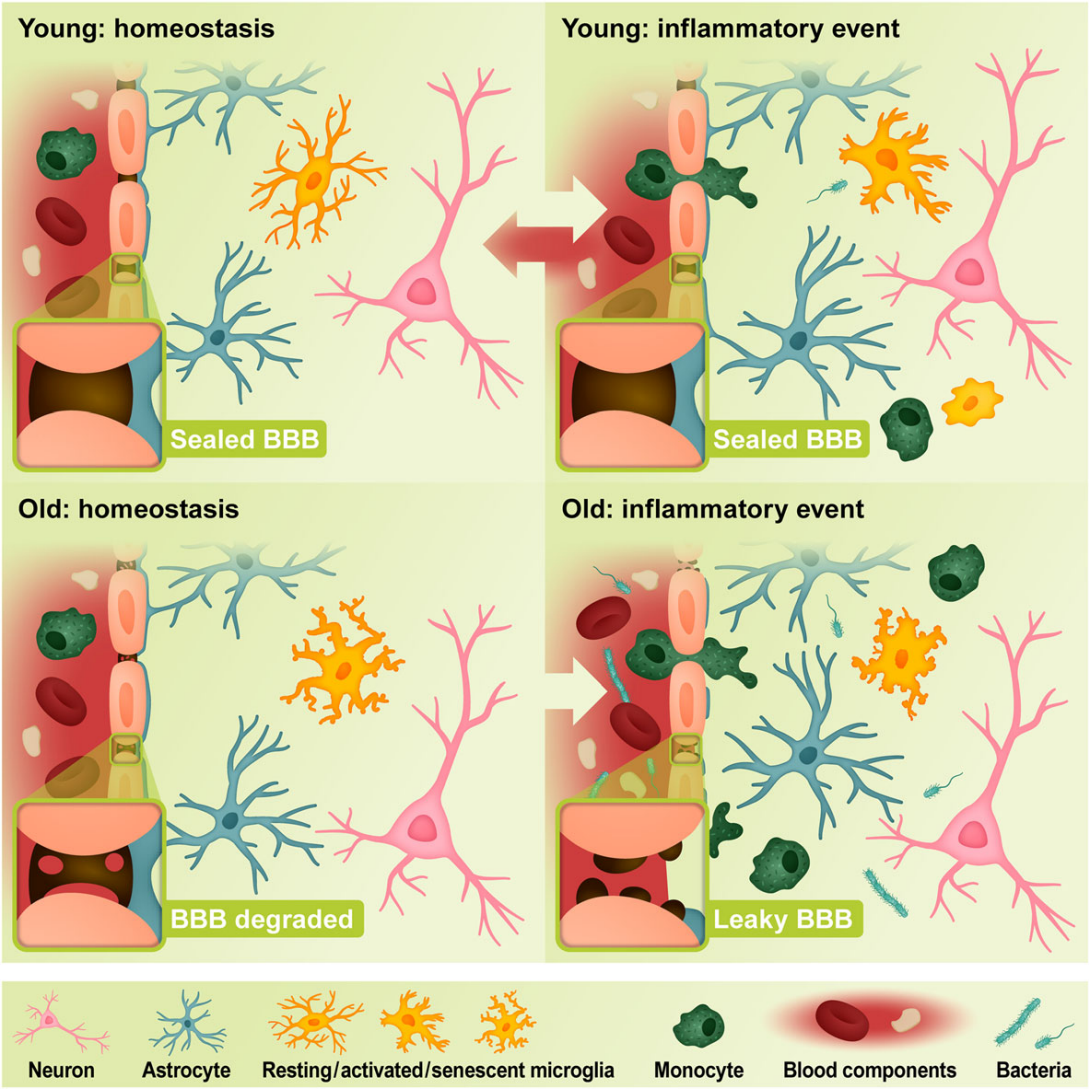
Inflammatory responses in the young and old brain. The BBB is intact in the young brain, reinforced by tight junctions, endothelial cells, pericytes, and astrocytes (blue). During homeostasis, microglia (orange) perform a surveillance role with extended processes. Microglia and astrocytes are activated by an inflammatory event, during which the microglia prepare for phagocytosis to eliminate the inflammatory stimulus, such as during a bacterial infection following an injury. Peripheral immune cells such as monocytes (green) are also able to cross the BBB to enhance phagocytosis and debris clearance in the brain. After the inflammatory event, microglia in the young brain are able to return to their surveillance state. After a lifetime of such events, however, microglia in the aged brain acquire a primed state, where they exhibit persistent low-level inflammation during homeostasis. During aging, the BBB also becomes leaky. Primed microglia are fast to react to inflammatory stimuli, but have a high risk of becoming senescent and unable to perform phagocytosis and clear infection. If the microglia are no longer able to remove the infection, they continue to release inflammatory mediators, leading to increased inflammatory cell migration past the BBB, which is further permeabilized during disease

A similar sequence of events occurs in the brain, which has its own resident immune cells, microglia. In the healthy brain, microglia participate in several housekeeping functions, including the maintenance of synapses (i.e., elimination, pruning or maturation), neurogenesis, the regulation of cognitive functions, and immunological surveillance. In the immune context, these cells sense pathological stimuli and inflammatory signals, produce inflammatory signals, and are involved in phagocytosis. Notably, recent studies have shown that microglia are highly heterogeneous across the different regions of the brain [3, 4]. In general, the number of microglial cells remains steady from late postnatal stages until old age due to the spatial and temporal coupling of proliferation and apoptosis [5], which occurs randomly throughout the brain [3]. At this stage, microglia are morphologically characterized by a small soma and ramified processes (Fig. 3). Upon activation, however, the self-renewal pattern shifts to selected clonal microglial expansion [3], in which activated cells acquire an amoeboid phenotype by shortening their cellular processes and enlarging their soma. At the molecular level, activated microglia upregulate the expression of many molecules (e.g., CD11b, Iba1, TLRs, CX3CR1, TREM2) and acquire antigen presentation features by expressing major histocompatibility complex-II (MHC-II), B7.1 and B7.2 (CD80/86). Proper priming of microglia during immune responses is tightly regulated by changes in the equilibrium of pro- and anti-inflammatory signals and is a fundamental step in the removal of pathogens and microbes by phagocytosis, as well as the clearance of toxic molecules, cell debris, remains of the extracellular matrix, myelin derivatives, and protein deposits (e.g., Aβ, α-synuclein). In the resolution phase, the excess microglia are removed by a dual mechanism of cell egress and apoptosis to re-establish the stable microglial network [3].

Similar to microglia, astrocytes play multiple roles in organizing and maintaining brain structure and function. Mounting evidence suggests that astrocytes dynamically modulate information processing, signal transmission, neural and synaptic plasticity, and blood-brain barrier (BBB) homeostasis. Their role in immune responses, however, remains poorly understood. Gene transcriptome analyses in the mouse brain have revealed that astrocytes are highly immune reactive and upregulate unique sets of genes that can either promote or hinder recovery depending on the immunological trigger. For instance, astrocytes seem to display a protective molecular phenotype during ischemic events, whereas their molecular phenotype seems to be harmful following *Escherichia coli* (*E. coli*) lipopolysaccharide (LPS)-nduced inflammation [6]. In the retina, astrocytes produce lipoxins that exert anti-inflammatory and neuroprotective effects against acute and chronic injury [7]. Recent evidence has indicated that production of the cytokine interleukin (IL)-33 by astrocytes is an important step in microglial synapse engulfment and neural circuit development [8]. Moreover, it has been shown that the pro-inflammatory cytokine tumor necrosis factor-α (TNF-α) signals through astrocytes to alter synaptic transmission and impair cognition in a mouse model of multiple sclerosis [9]. Although much of the detail of astrocyte heterogeneity remains elusive, it has been proposed that activation of the transcriptional factors, signal transducer and activator of transcription 3 (STAT3) and nuclear factor-κB (NF-κB), is at least in part related to their protective [10] and detrimental [11] phenotypes, respectively. Clearly, additional studies of the role of astrocytes in inflammation and immune regulation will have a tremendous impact in advancing our understanding of brain function during healthy and disease states.

## The effect of aging on microglia

Aging is the most prevalent risk factor for AD. As individuals age, a gradual deterioration, known as immunosenescence, occurs in the peripheral and central components of immune system, raising susceptibility to infections and diseases. In the aged human brain, altered expression of several immune-related genes has been observed in distinct regions, a pattern that is exacerbated in AD [12]. Age-related changes in the brain have also been observed at the cellular level, with microglia showing substantial phenotypic changes. Grabert and colleagues have recently shown through genome-wide transcriptional profiling that aging impacts microglia in a non-uniform and region-dependent manner in mice. More importantly, they discovered that changes in immune-related genes, and to lesser extent bioenergetics-related genes, are largely associated with the region- and age-related diversity of these cells [4]. Microglia isolated post-mortem from aged human parietal cortex also show a distinct transcriptomic profile versus cells obtained from young subjects, in which genes associated with cell adhesion, axonal guidance, cell surface receptor expression, and actin assembly are particularly affected [13]. Morphologically, aged microglia from human cerebral cortex exhibit several abnormalities, including deramification, spheroid formation, gnarling, and fragmentation of processes (Fig. 3) [14].

Microglia are also highly affected by aging and disease at the molecular level. Using a newly developed high-dimensional single-cell proteomic mapping, Mrdjen and colleagues identified distinct populations of inflammatory cells, including microglia, in the adult mouse brain. Notably, they were also able to detect robust changes in the proteomic signature of these cells during aging, neurodegeneration and neuroinflammation [15]. Compared to cells from young mice, a subset of aged microglia expressed high levels of the phagocytosis-associated markers CD11c and CD14. Moreover, distinct populations of microglia were identified in the aged mouse brain, in which the reactive population expressed higher levels of CD11c, CD14, CD86, CD44, programmed death-ligand 1 and MHC-II, and lower levels of the microglial homeostatic checkpoint markers CX3CR1, MerTK (C-MER proto-oncogene tyrosine kinase), and Siglec-H, compared to the non-reactive microglia [15]. Biochemically, aged microglia produce more reactive oxidative species and inflammatory cytokines [16], and they have also been found to suppress neural precursor activity in the mouse hippocampus [17]. TNF-α, IL-1β, and IL-6 are among the pro-inflammatory cytokines that are overproduced during aging, an effect which is thought to be caused by overstimulation of the transcription factor NF-κB when microglia undergo cellular senescence [12].

The overproduction of pro-inflammatory mediators leads to the sensitization of microglia, or age-related microglial priming (Figs. 2 and 3), whereby aged microglia produce an exaggerated, but inefficient, response to inflammatory stimuli. Wendeln and colleagues recently demonstrated that peripheral stimulation of mouse microglial cells by repeated LPS injection can cause epigenetic changes in these cells for up to six months [18]. This suggests that the baseline level of inflammation may increase upon repetitive inflammatory stimuli, potentially the cause of microglial priming. Such cumulative and long-lasting changes in the equilibrium of inflammatory mediators worsen the ability of microglia to perform basic physiological functions and are likely a contributing factor in neurodegenerative processes. For instance, young microglia can more efficiently phagocytose disease-related proteins, such as Aβ [19] and α-synuclein [20], than aged microglia. Likewise, it has been shown that aged microglia have impaired motility, surveillance, and phagocytosis responses to demyelinating lesions [21], in part due to reduced lysosomal function [22]. Taken altogether, these studies suggest that the susceptibility of different brain regions to AD may be linked to the overstimulation of immune-related signals during aging and the detrimental impact of these cascades in the functionality of the distinct populations of microglia present in each brain region.

## The opening of the blood-brain barrier during aging

Another characteristic of aging with relevance for AD is the increased permeability of the BBB to immune cells and molecules from peripheral tissues. Loss of BBB integrity seems to occur before hippocampal atrophy [23, 24] and cognitive impairment [25], suggesting that this breakdown precedes the neurodegenerative process in AD. This evidence also indicates that peripheral activation of the immune system can contribute to the impairment in brain function and neurodegenerative processes that occur in AD [26]. Interestingly, an association has recently been made between midlife inflammation and late-life brain volume. Compared to individuals with no elevated midlife inflammatory markers, individuals with elevations in 3 or more markers had, on average, 5% smaller hippocampal and AD signature region volumes [24].

In healthy individuals, the cellular components of the neurovascular unit, including endothelial cells, pericytes and astrocytes, orchestrate the formation of tight junctions in endothelial cells in response to inflammatory signals to limit the infiltration of cells and molecules from the periphery. A secondary protective mechanism can also be triggered in astrocytes, which upon opening of endothelial tight junctions can regulate leukocyte and humoral transit by forming tight junctions of their own at the glia limitans of the BBB. Inhibiting the formation of this inducible astrocytic barrier increases disease severity in mouse models of neuroinflammation [27]. Therefore, a possible mechanism for the impairment in the BBB function is the immune-related senescence of its cellular components.

Endothelial cells, pericytes and astrocytes at the BBB are particularly vulnerable to the effects of aging and chronic stimulation by inflammatory mediators (Fig. 3). During aging, mouse brain endothelial cells express higher levels of TNF-α and decreased expression of the tight junction proteins occludin-1 and zonula occludens-1, which correlates with increased peripheral inflammation [28]. Aging and, more aggressively, AD also trigger damage to pericytes as demonstrated by the increased levels of platelet-derived growth factor receptor-β observed in mouse models [23]. Although the mechanism inducing pericyte injury is still unknown, Bell and colleagues have shown that the age-dependent vascular damage in pericyte-deficient mice precedes neuronal degenerative changes, learning and memory impairment, and the neuroinflammatory response [29].

In astrocytes, transcriptome analysis has revealed that aging induces an upregulation of several immune-related genes [30]. An age-dependent change in astrocyte phenotype was identified by comparing gene expression in astrocytes from 10-week- and 2-year-old mice using RNAseq. This study demonstrated that astrocytes from healthy 2-year-old mice expressed genetic markers which correspond to the activated A1 phenotype, including C4a, C3, Serpina3n, and Cxcl10 [31]. Mechanistically, it has been shown that IL-1β suppresses the astrocytic expression of sonic hedgehog [32], a protein that protects the BBB by upregulating tight junction proteins in capillary endothelial cells [33]. IL-1β also increases the production of pro-inflammatory chemokines such as CCL2, CCL20, and CXCL2 by astrocytes, which induce immune cell migration from the periphery, and exacerbate BBB disruption and neuroinflammation [32]. Hence, an excessive pro-inflammatory phenotype significantly disrupts the protective role of astrocytes in maintaining BBB integrity.

Inflammation and aging are therefore closely linked with studies suggesting that low levels of inflammation correlate better with healthy brain function [24] and longevity [34]. Considering that most cells in the brain, including astrocytes [35] and microglia [36], have a long life-span, it is plausible that the build-up and overstimulation of inflammatory signals trigger multiple cumulative molecular modifications (e.g., telomere shortening, DNA damage, epigenetic modifications, lysosomal dysregulation) that eventually contribute to cellular senescence and loss of function. This idea is, at least in part, supported by a repopulation study in a mouse model of neurodegeneration, which demonstrated that, following pharmacological-induced cell depletion, the microglia that repopulated the brain displayed the morphological phenotype of young cells. Remarkably, the animals also showed significant improvement in brain functions [37]. Whether cell repopulation methods can reset the molecular signatures of immunosenescence is still unknown. Additional studies are needed to clarify the underlying cellular and molecular mechanisms related to the immune dysregulation that occur during aging which divert individuals from the relatively benign process of normal brain aging to the pathological processes associated with AD.

## The production of Aβ as a physiological component of immune responses

Accumulation of Aβ is without doubt one of the major triggers of neurodegeneration in AD. Evidence suggests, however, that Aβ acts as a physiological trigger of pro-inflammatory responses during healthy aging and that its build-up results from age-related defects in the immune signals that control the molecular mechanisms involved in the production, degradation and clearance of this peptide. At picomolar to nanomolar concentrations, Aβ modulates synaptogenesis, axonal growth and guidance, synaptic plasticity, oxidative stress, learning, and memory [38]. Moreover, at higher concentrations, Aβ binds to inflammatory receptors (i.e., TLR2, TLR4, RAGE) [39–41], activates immune-related transcriptional factors (i.e., NF-κB) [42, 43], produces inflammatory mediators (i.e., TNF-α, COX-2, iNOS) [42, 44], and exerts potent antimicrobial activity [45], indicating that it is upregulated as part of the physiological acute innate immunity.

As the synthesis of Aβ is dependent upon sequential β-secretase-1 (BACE1) and γ-secretase cleavage of amyloid precursor protein (APP), any factor which influences the levels of these proteins could potentially contribute to the accumulation of Aβ (Fig. 1). Importantly, responsive sites for immune-related transcriptional factors which are commonly over-activated during aging and AD, including NF-κB, peroxisome proliferator-activated receptor-γ (PPAR-γ), and STAT-1, have been described in the regulatory promoter region of the genes controlling the expression of APP [46], BACE1 [47–49], and proteins of the γ-secretase complex [50]. Bourne and colleagues have proposed that *BACE1* transcription is repressed by NF-κB in neurons but activated in reactive astrocytes [47]. Although neurons account for most BACE1-mediated Aβ synthesis in AD, it has been demonstrated that inflammatory mediators, such as IFN-γ, TNF-α, and IL-1β, induce the expression of BACE1 and the release of Aβ in astrocytes, suggesting that these cells also contribute to the amyloidosis in AD [48, 51]. Moreover, He and colleagues have shown that genetic deletion of the TNF type-1 death receptor (TNFR1) in the APP23 mouse model leads to reduced NF-κB-mediated *BACE1* expression, which is associated with lower Aβ levels and improved learning and memory [52]. The brain steady-state levels of BACE1 and Aβ are also increased by the inflammatory enzyme 12/15-LOX [53]. The mechanism whereby 12/15-LOX controls BACE1 has not been elucidated; however, this enzyme modulates the levels of free fatty acids and eicosanoids that are known to regulate PPAR-γ activity during inflammatory conditions [54]. Notably, PPAR-γ acts as a suppressor of BACE1 expression [55]. Another member of the LOX family, 5-LOX, has also been shown to activate γ-secretase and induce Aβ production, and levels of 5-LOX are increased in the brain during aging [56]. The relevance of the immune-related signals in the regulation of distinct components of the amyloidogenic cascade reinforce the idea of Aβ as a mediator of the innate immune system. Likewise, these studies indicate that chronic inflammation is a potential contributor to the overproduction of Aβ in AD. However, whether the age-dependent increase of inflammation can trigger the excessive production of Aβ on its own to promote the onset of AD requires further investigation.

## The proteases involved in the proteolysis of Aβ are also linked to inflammation

As for any major inflammatory cascade, many physiological cellular processes are in place to regulate the availability of Aβ in the brain. For instance, neurons, microglia, and astrocytes produce the protease neprilysin to counter-regulate the increase in Aβ levels. Although the participation of neprilysin in the brain immune response has not been explored, data from peripheral models indicate that it is an important component in the resolution of responses involving other inflammatory peptides. Genetic deletion of neprilysin promotes spontaneous inflammatory edema that is caused by diminished degradation of the pro-inflammatory neuropeptides substance P and bradykinin [57]. Blockage of neprilysin activity also exacerbates colitis [58], ileitis [59], and dermatitis [60], and recombinant neprilysin ameliorates inflammation [58]. Other proteases, including insulin-degrading enzyme (IDE) and matrix metalloproteinases (MMPs), are also produced upon activation of glial cells to neutralize Aβ. Kong and colleagues have shown that Aβ-induced IDE upregulation is mediated by the activation of β_2_-adrenergic receptors in microglia [61], whereas this process is regulated by the low-density lipoprotein receptor-related protein-1 (LRP-1) in astrocytes [62]. IDE activity has also been linked to the degradation of the inflammatory chemokine macrophage inflammatory protein-1 in microglia [63]. In addition, astrocytic LRP-1 regulates the levels of MMP-2 and MMP-9 in response to Aβ, and inhibition of LRP-1, MMP-2 and MMP-9 in these cells results in accelerated Aβ accumulation in the APP/PS1 mouse model of AD [62]. The appropriate activation of these degrading enzymes is therefore required for the resolution of Aβ-mediated immune responses and the prevention of chronic inflammation. Importantly, the overall brain activity of neprilysin and IDE are reduced during aging and AD [64, 65]. On the other hand, MMP-9 seems to be overproduced in response to Aβ, thereby contributing to the damage and leakage of the BBB [66].

## The immune-related clearance mechanisms for Aβ

Other cellular mechanisms that terminate Aβ responses include clearance through phagocytosis and intracellular degradation, and transcytosis across the BBB. These processes involve the binding of Aβ to transmembrane proteins such as LRP-1 and members of the ATP-binding cassette (ABC) transporter family. LRP-1 is an endocytic and cell-signaling receptor, and is important for the uptake of Aβ by astrocytes [62], neurons [67], and endothelial cells [68]. Moreover, during immune responses, LRP-1 expressed in macrophages and microglia acts as a scavenger receptor, removing debris, and necrotic and apoptotic cells [69]. The stimulation of this receptor also regulates inflammatory pathways in immune cells [70]. LRP-1 agonists suppress the expression of pro-inflammatory mediators, whereas its antagonists increase the expression of pro-inflammatory signals through the activation of c-Jun N-terminal kinase and NF-κB [71, 72]. Therefore, activation of LRP-1 in microglia keeps these cells in an anti-inflammatory and neuroprotective status during inflammatory responses. Studies in human aged and AD subjects indicate that brain LRP-1 expression decreases and inversely correlates with the age of onset of AD [73]. It has also been reported that LRP-1 expression is reduced in the microvasculature of the BBB in aged rats [74]. This decline in LRP-1 levels might be another contributing factor to the overall increase in inflammation and Aβ accumulation in the brain during the aging process.

ABCA1, ABCA7, and ABCB1 (also known as P-glycoprotein) are members of the ABC family of transporters that are important for the clearance of Aβ, and hence, their loss of function results in the accumulation of plaques in the brain. ABCA7 is particularly relevant, as recent studies have shown that loss-of-function polymorphisms at its gene increase the risk for late- [75] and early-onset AD [76]. These transporters use the binding and hydrolysis of ATP to power the translocation of several substrates ranging from ions to macromolecules across membranes. For this reason, their normal physiological activity is required to maintain the healthy balance of mediators in the brain. Accordingly, Karasinska and colleagues have shown that acute LPS induces augmented brain pro-inflammatory response in mice lacking the *ABCA1* gene, suggesting that this protein participates in the resolution of immune responses by facilitating the clearance of inflammatory mediators [77]. Moreover, the expression of ABC transporters in immune cells contributes directly to phagocytosis. ABCA7-mediated activation of extracellular signal-regulated kinase (ERK) enhances the phagocytosis of apoptotic cells and the complement protein C1q by macrophages [78]. Similarly, stimulation of ABCA1 results in the upregulation of the phagocytic proteins multiple epidermal growth factor-like domains 10 (MEGF10) and engulfment adapter PTB domain containing 1 (GULP1) in reactive astrocytes, which is important for the engulfment of debris during pathological processes [79]. ABCA7 also participates in the phagocytosis of Aβ by microglia [80]. Interestingly, the overall expression levels of ABCA1 and ABCA7 are increased in AD [81, 82], indicating that their function is impaired in the diseased brain. Additional studies are needed to elucidate whether excessive and chronic inflammation can obstruct the activity of these transporters, causing a decline in Aβ clearance from the brain.

## Microglial phagocytosis decreases during aging

As the major phagocytic cells in the brain, microglia have a central role in the clearance of Aβ. However, the efficacy of this removal diminishes during aging, and particularly in AD [19, 83]. Despite their inability to clear Aβ, microglia continue releasing pro-inflammatory mediators to further stimulate the immune response [84], thereby creating a vicious cycle which leads to the build-up of activated immune cells, inflammatory mediators, and Aβ. This process can be reversed by blocking Aβ synthesis [85], and it is partially caused by the senescence of microglia [86]. Microglia from old APP/PS1 mice exhibit lower expression of the Aβ-binding scavenger receptors scavenger receptor A (SRA), CD36, and receptor for RAGE than observed in cells from young mice. In contrast, these microglia express higher levels of the pro-inflammatory cytokines IL-1β and TNF-α, suggesting that there is an inverse correlation between pro-inflammatory cytokine production and Aβ clearance. This idea is supported by in vitro studies in which treatment of microglia with TNF-α resulted in reduced SRA and CD36 expression, and Aβ uptake [83]. In AD mouse models, microglia also display substantial impairment in calcium signaling [87] and beclin-1-mediated recycling of the phagocytic receptors CD36 and Trem2 [88], which are linked to poor Aβ internalization. Based on longitudinal human brain imaging studies, Fan and colleagues have postulated that the status of microglia activation shifts from an early protective phenotype to a late harmful phenotype during the progression of AD [89]. This change in the overall microglial phenotype could be associated with the chronic activation of distinct populations of microglia, which are either CX3CR1^+^ or Trem2^+^ and release inflammatory mediators or perform Aβ phagocytosis, respectively [90].

Although strong evidence indicates that aging impairs microglial activity, our understanding of the complex relationship between microglial senescence, Aβ, and AD is still incomplete, as some studies have shown that microglial phagocytic activity towards Aβ might not necessarily relate to changes in neurotoxicity and cognition. Investigations using the J20 APP mouse model have revealed that inhibition of microglial phagocytic activity by minocycline before Aβ accumulation results in increased amyloid plaque load, reduced inflammation, and improved cognitive performance, indicating that chronic inflammation can disrupt neuronal function independently of Aβ. However, when microglial inhibition was performed after Aβ deposition had begun, inflammation was suppressed by minocycline with no effect on plaque load or improvement in cognitive performance [91]. Adding to the complexity of the inflammation to AD network, pharmacological or genetic depletion of microglia following robust Aβ accumulation does not change the plaque levels but does rescue dendritic spine loss, prevent neuronal loss, and improve cognitive performance [92–95].

## The complexity of pro- and anti-inflammatory timing

Considering the strong evidence linking inflammation to the accumulation of Aβ in AD, many research groups have been using loss- and gain-of-function approaches to identify the role and potential therapeutic value of specific mediators involved in the activation and resolution phases of inflammation. However, this has not proven to be a simple task, as recent studies have reported opposite neuropathological effects by manipulating pro- and anti-inflammatory signals in models of Aβ pathology. For example, genetic deletion of CX3CR1 in AD mouse models changes the inflammatory milieu, resulting in higher microglia-mediated Aβ phagocytosis [96] and reduced neuronal loss [97], suggesting that modulating the production of inflammatory signals is beneficial in AD. Similarly, genetic deletion of the inflammatory enzymes caspase-1 or NLR family pyrin domain containing 3 (NLRP3), which are involved in the synthesis of IL-1β, improves clearance of Aβ by microglia and cognitive performance in AD mice [98]. Fu and colleagues have shown that APP/PS1 mice treated with the cytokine IL-33 present lower levels of pro-inflammatory gene expression (i.e., *IL-1β*, *IL-6*, *NLRP3*) in association with reduced Aβ load, increased magnitude of long-term potentiation (LTP) at Schaffer collateral-CA1 synapses, and improved cognitive function [99]. Blockage of mediators of the complement cascade, including C1q [100], C3 [101, 102], and C5a [103], also confers neuroprotective effects in mouse models of AD. Moreover, deficiency of IκB kinase β, which activates NF-κB, in microglia reduces inflammatory activation and Aβ load in the brain of TgCRND8-APP mice, effects which are associated with a reduction in cognitive deficits and preservation of synaptic structural proteins [104]. Overall, these studies suggest that blockage of pro-inflammatory responses is beneficial in AD. However, the integration between inflammation and AD is not so simple, as it has been demonstrated that stimulation of pro-inflammatory TLR4 with monophosphoryl lipid A (MLA) results in Aβ phagocytosis by microglia in vitro, as well as a decrease in Aβ load and cognitive impairment in the APP/PS1 mouse model [105]. Interestingly, although MLA is at least 100-fold less pyrogenic than the gram-negative bacterial cell wall constituent LPS, it maintains many of the immunomodulatory properties of LPS [106]. Based on this evidence Michaud and colleagues have suggested that the age-related defects in microglia can be overcome with a pro-phagocytic, yet mildly pro-inflammatory, phenotype leading to the improved clearance of Aβ [105].

Targeting of anti-inflammatory and pro-resolution mediators has also produced mixed results, with the activation of IL-10 anti-inflammatory signaling in AD mouse models resulting in impairment in Aβ phagocytosis and exacerbated AD neuropathology [107, 108]. On the other hand, stimulation of anti-inflammatory LXA_4_ [109, 110] or prostaglandin EP4 receptor (EP4) [111] signaling facilitates microglia-mediated Aβ clearance and reduces AD-like pathology in mice. Notably, whereas IL-10 is increased [112], the levels of LXA_4_ [113] and EP4 [111] are reduced in human AD, indicating that targeting missing inflammatory mediators may be a better approach to restore the balance between pro- and anti-inflammatory signals in the immune responses during aging and AD.

Taken altogether, these studies illustrate the complexity of targeting distinct inflammatory mediators in AD, but also clearly demonstrate that the phagocytic activity of microglia is highly important in the clearance of Aβ deposits. Consequently, one of the current challenges in the field is to identify the underlying molecular mechanisms that regulate the phenotype of microglia or distinct microglial populations in order to develop strategies that stimulate the protective phagocytic phenotype while inhibiting the detrimental pro-inflammatory phenotype.

## Chronic inflammation causes tau phosphorylation and worsens pathology

Neurons express many inflammatory receptors and molecules, including complement, MHC-I, TNFR1, IL-1R, and TLRs, allowing them to interact directly with microglia [114]. Inflammatory signals can consequently directly activate neuronal protein kinases and phosphatases, such as cyclin-dependent kinase 5 (CDK5), glycogen synthase kinase-3β (GSK3β), ERK, and protein phosphatase 2A (PP2A), that regulate tau phosphorylation and neuronal microtubule assembly [115–118]. Although the physiological relevance of this regulation remains to be elucidated, growing evidence suggests that it might be important for the regulation of synaptic pruning, LTP, neurogenesis, and cognitive function mediated by the activation of immune-related cascades in neurons. Thus, not only does tau appear to have a role in the coordination of neuron-microglia communication during the physiological activation of immune cascades in the brain, but age-related chronic inflammation also has the potential to trigger the onset and progression of tau pathology in AD.

Although there has been extensive research into Aβ and inflammation, the effects of inflammation on tau pathology and vice versa remain comparatively untouched. It seems clear, however, that chronic microglial activation and inflammation cause the propagation of pathological tau species and participate in tau-induced neurotoxicity [119–122]. With regards to how inflammation induces tau pathology, most studies have shown that chronic levels of inflammatory mediators exacerbate the activation of key protein kinases that control the phosphorylation levels at tau. Chronic release of TNF-α from microglia, for instance, has been shown to induce the aggregation of tau in neurons in vitro [123]. On the other hand, blockage of microglia with minocycline reduces the inflammatory response and propagation of tau pathology in the hTau mouse model [124]. Inhibition of inflammation by arginase-1 overexpression, which counterbalances the activity of nitric oxide synthases, facilitates autophagy and decreases tau pathology in the rTg4510 mouse model of tauopathy [125]. Roe and colleagues have shown that systemic stimulation of TLR4 by LPS promotes acute GSK3β- and CDK5-dependent phosphorylation of endogenous murine tau. It has been suggested that this process may be important for stress-induced responses, as it can be blocked by the genetic deletion of corticotropin-releasing factor receptor [126]. Notably, genetic deletion of tau decreases the neurotoxicity and levels of inflammatory proteins IL-1β and neuron-derived alarmin caused by LPS in mice [121]. The authors of this study proposed that this phenomenon is likely associated with the indirect reduction of microglial activity due to the diminished production of pathological tau species. Brain stimulation of TLR4 in the rTg4510 mouse model also results in the activation of microglia and the phosphorylation of tau [127]. In the 3xTg-AD mouse model, which develops both Aβ and tau pathologies, chronic treatment with LPS results in CDK5-dependent tau phosphorylation without affecting Aβ levels in adult animals (~ 6 months of age). Tau phosphorylation is observed at Ser202/Thr205 and Thr231/Ser235, but not Ser396/Ser404, which are recognized by AT8, AT180, and PHD finger protein 1 (PHF-1) antibodies, respectively [115]. Interestingly, activation of TLR4 induces tau pathology more aggressively in older 3xTg-AD mice (~ 12 months of age), indicating that aging increases the susceptibility of tau to inflammatory events. Aged 3xTg-AD mice chronically treated with LPS show tau phosphorylation at AT8, AT180 and PHF-1 epitopes, tau aggregation into NFTs and cognitive impairment, but no change in Aβ plaque load. In these older animals, however, modulation of tau pathology by TLR4 is mostly mediated by GSK3β [128]. Although no mechanism has been proposed for this age-dependent effect, it is possible that the exacerbated accumulation of pathological forms of tau is caused by the impairment of its intracellular clearance mechanisms such as autophagy and proteasome [129].

## The diverging impact of inflammation on Aβ and tau pathology

Adding to the complex connection between inflammation and AD is the mounting evidence that inflammation can exert opposing effects on Aβ and tau. It has been shown that activation of IL-1β signaling triggers the activation of kinases CDK5, GSK3β, and p38 mitogen-activated protein kinase (p38-MAPK), resulting in tau hyperphosphorylation and cognitive impairment in the 3xTg-AD mouse model [130]. Mechanistically, aging seems to cause a decrease in the levels of the senescence-related protein sirtuin 1 in microglia. This results in excessive IL-1β production, which in turn causes tau pathology and cognitive deficits [131]. Overexpression of IL-1β in the 3xTg-AD mouse model, however, promotes opposing effects on the amyloid and tau pathologies by accelerating the accumulation of pathological forms of tau while reducing the overall levels of Aβ plaques. Stimulation of IL-1β results in an increase in the number of activated microglia surrounding Aβ plaques, but also promotes the activation of GSK3β and p38-MAPK, leading to a higher level of tau phosphorylation [132].

Additional studies have reinforced the idea that inflammation can have distinct effects on these two major AD pathological markers. For instance, as discussed above, CX3CR1 deficiency in mouse models of amyloidosis mitigates Aβ accumulation by altering microglial activation and promoting microglial phagocytosis [96, 133]. On the other hand, blockage of CX3CR1 signaling increases IL-1β/p38-MAPK-mediated tau phosphorylation in the hTau model of tauopathy [117]. Genetic deletion of membrane-anchored CX3CL1, which acts as a CX3CR1 agonist, in the APP/PS1 mouse model also reduces Aβ deposition through the increase in microglia-mediated phagocytosis while inducing neuronal tau phosphorylation [134].

Moreover, deletion of progranulin, which has been extensively associated with frontotemporal dementia [135], results in increased TYRO protein tyrosine kinase binding protein (TYROBP) signaling activation and microglial Aβ phagocytosis in the APP/PS1 mouse model, whereas it increases tau pathology in human P301L tau-expressing mice [136]. These opposing immune signal-induced effects on Aβ and tau accumulation raise significant concerns regarding the clinical efficacy of therapies designed to inhibit or activate inflammation in AD, particularly considering that tau levels better correlate with the cognitive deficits observed during the disease process [137]. Although challenging, developing strategies that can modulate the immune system by favoring Aβ clearance while reducing the levels of inflammatory signals that drive tau pathology will likely produce better clinical outcomes.

## Chronic inflammation impairs cognition and synaptic plasticity

Although learning and memory, synaptic plasticity, and inflammation are usually considered separately, there is convincing evidence that inflammatory pathways have a significant role in the development and maintenance of the health and plasticity of the central nervous system (CNS) and are not only activated during times of defense and disease. Physiologically, immune-related receptors such as MHC-I, TNFR1, and IL-1R participate in LTP and modulate learning and memory formation [114]. For example, neurons from MHC-I-knockout mice show increased synaptic plasticity, excitability, and LTP [138], which is increased in 12-month old mice [139], likely exerting this effect via modification of NMDAR and α-amino-3hydorxy-5-methyl-4-isoxazolepropionic acid receptor (AMPAR) function and trafficking [140]. It has recently been shown that IL-1β directly suppresses hippocampal plasticity via neuron-specific mechanisms [141] and that increased pro-inflammatory IL-1 accessory protein signaling, specifically at the synapse, underlies the augmented vulnerability to IL-1β-mediated cognitive impairment that occurs with age [142]. Moreover, immune-related cascades contribute to plasticity through the regulation of microglia-mediated synaptic pruning. It has been shown that genetic depletion of microglia in CX3CR1^CreER/+^-R26^iDTR/+^ mice results in reduced synapse formation and memory deficits [143]. A similar effect was found in CX3CR1-knockout mice, in which neural connectivity was reduced together with a reduced behavioral tendency to prefer social interaction [144]. Mechanistically, synaptic pruning can be regulated by inflammatory signals, including the complement cascade factors C1q-C3R [145] and chemokines CX3CL1-CX3CR1 [146]. Hence, it is not surprising that excessive neuroinflammation can lead to direct impairment of cognitive function. During AD, there is evidence that microglia cause a significant amount of synaptic loss, likely through dysfunctional synaptic pruning [95, 147]. Furthermore, chronic neuroinflammation leads to the loss of synaptic-associated proteins, thereby causing neuronal damage [148]. Thus, chronic inflammation influences each of the three hallmarks of AD pathology: Aβ accumulation, tau phosphorylation and cognitive decline associated with synaptic and neuronal loss.

## The genetic risk factors for AD are involved in the inflammatory response

### The ApoE isoforms and their relevance to inflammation

Apolipoprotein E (ApoE), the most prevalent genetic risk factor for late-onset AD, is a polymorphic lipoprotein which mediates the transport and delivery of cholesterol and other lipids through cell surface ApoE receptors. Human ApoE exists in three isoforms in which polymorphisms are found in the receptor binding domain—ApoE4 (R112, R158), E3 (C112, R158), and E2 (C112, C158)—with ApoE4 conferring the highest risk for AD. ApoE has a variety of roles in the CNS, including lipid homeostasis, repair of injured neurons, maintenance of synaptic-dendritic connections, and scavenging toxins including Aβ. Notably, ApoE is also an important modulator of immune responses, as evidenced by greater immune activation in mice lacking ApoE [149]. Studies in knockout mice have mostly demonstrated that ApoE has an anti-inflammatory effect, as seen in models of ischemia [150], traumatic brain injury (TBI) [151], and chemical-induced neuroinflammation [152].

In the brain, the inflammatory processes mediated by microglia and astrocytes are altered in an isoform-specific manner, with ApoE4 promoting the strongest pro-inflammatory effects [153, 154] and ApoE4 transgenic mice being more susceptible to inflammation than those expressing ApoE2 and ApoE3 [155–157]. Interestingly, studies in humans have shown that intravenous administration of LPS in ApoE3/ApoE4 patients produces higher hyperthermia and plasma TNF-α levels and earlier plasma IL-6 than in ApoE3/ApoE3 subjects [158]. Likewise, animals expressing ApoE4 show greater levels of pro-inflammatory cytokines and neurotoxicity in response to systemic [159] and central [160] LPS, than their ApoE3 counterparts. Together, these studies indicate that ApoE4 has lost its anti-inflammatory properties and inefficiently prevents the detrimental impact of inflammation. The mere expression of ApoE4 can therefore potentiate the immune activation associated with aging, eventually leading to neurodegenerative disease. Supporting this idea, studies in mice and humans have demonstrated that ApoE4 amplifies the pro-inflammatory innate immune response induced by Aβ, resulting in a robust inflammatory phenotype that causes neuronal dysfunction [156, 161]. In addition, antagonism of the liver X receptor in APP23 mice leads to increased microglial activation and phagocytosis of Aβ, effects not observed in the absence of ApoE, indicating that ApoE is essential for the microglial response to Aβ [162]. Interestingly, ApoE is also an endogenous ligand of TREM2 [163], and their interaction appears necessary to mediate the switch from a homeostatic to a neurodegenerative microglial phenotype after phagocytosis of apoptotic neurons, as observed in microglia associated with Aβ plaques [164]. Whereas ApoE3 has an essential anti-inflammatory role in AD and other diseases, the balance between pro- and anti-inflammatory signaling is aberrantly altered for those carrying ApoE4 [154]. Although the reason for this is yet to be fully elucidated, the interaction between ApoE and TREM2 may shed some light on the different mechanisms caused by ApoE isoforms, with respect to inflammation.

### The TREM2 mutations that lead to changes in microglial phagocytosis and increased AD risk

TREM2 is a relatively new addition to the list of genes which are reported to be related to an increased risk of developing AD, with a genome-wide association studies (GWAS) in 2013 confirming that R47H TREM2 mutant is associated with AD [165] and specifically tau hyperphosphorylation [166]. Although the R47H mutation has the strongest association with AD, patients have a significantly higher chance of mutation in their TREM2 exon 2 sequence than healthy controls. These mutations include Q33X, T66M and Y38C, which generally confer a loss of receptor function [165]. This finding is interesting given that TREM2 is necessary for microglial phagocytosis [167]. TREM2 acts with an adapter TYROBP (also known as DAP12) to detect stimuli, such as LPS and bacteria [168], and initiate phagocytic pathways within microglia via its immunoreceptor tyrosine-based activation motif [169]. Recently published research has also shown that TREM2 is a direct receptor for Aβ, inducing microglial depolarization and an increase in their pro-inflammatory response [170]. The TREM2-TYROBP complex has been shown to have both a pro- and anti-inflammatory role in the peripheral immune system; in dendritic cells, it has been demonstrated that *TYROBP* knockout can reduce pro-inflammatory chemokine production [171], and knockout of TREM2 can enhance the TLR response [172]. These two opposing findings indicate that the TREM2-TYROBP complex is a key, albeit complicated, player in the immune response, whereby the knockout of individual components may result in different effects. These data suggest that TREM2 subdues the activity of other inflammatory receptors, while TYROBP is responsible for pro-inflammatory TREM2-TYROBP signaling. Following proteolytic cleavage of TREM2 by a disintegrin and metalloproteinase (ADAM) proteases, extracellular soluble TREM2 (sTREM) activates microglia via the Akt-GSK3β pathway, leading to the activation of the NF-κB transcription factor and thus the transcription of pro-inflammatory cytokines through binding of microglial cell surface receptors [173]. Interestingly, the level of sTREM is elevated in the cerebrospinal fluid and plasma in neuroinflammatory diseases including AD, correlating with overall patient inflammation [174, 175].

As microglial phagocytosis has been shown to significantly reduce the plaque load observed in mouse models of AD, researchers investigated whether the TREM2 mutations associated with AD conferred a loss of receptor function, finding that the rare TREM2 variants caused impairment of phagocytosis [176]. The missense mutation R47H of TREM2 is associated with AD risk by dysregulating neuroinflammation and increasing AD pathology [165, 177]. Mazaheri and colleagues showed that TREM2 knockout reduced microglial reactivity and consequently blocked essential defense functions of microglia during disease progression [178]. They further showed that TREM2 knockout mice had impaired microglial migration and process outgrowth, likely due to dysregulation of genes associated with chemotactic motility. Recently, a TREM2^R47H^ mouse model has been produced, which displays abnormal macrophage apoptosis and necrosis [179]. Interestingly, these mice also failed to mount a pro-inflammatory response to challenges, including LPS, demonstrating a failure to produce pro-inflammatory cytokines. This further suggests that the R47H mutation of TREM2 renders it inactive. In support of these findings, it has also been shown that the uptake of ApoE-Aβ complexes is reduced in macrophages from human subjects carrying the R47H TREM2 variant [180]; however, whether ApoE isoforms affect TREM2-mediated neuroinflammation response in an isoform-specific manner in the pathogenesis of AD is still unknown.

Several studies over the past 3 years have investigated the relationship between Aβ and TREM2 activity by overexpressing TREM2 or using TREM2 knock out strategies. These studies have reported varying effects, depending on the mouse model and age of the animals, but may suggest similar trends. For example, in the absence of TREM2, mouse models of amyloidosis show that Aβ plaque load is decreased in younger mice, but increased in older animals [181]. Jay and colleagues suggest that this may be due to the changes in the microglial phenotype that occur during aging, perhaps due to the acquired senescence of microglia towards the later stages of AD [181]. When TREM2 was overexpressed via intracerebral lentiviral particle injection into either the hippocampus or cortex of 7-month-old APP/PS1 mice, a decrease in Aβ plaque load was observed in both brain regions 2 months later [182]. This translated to improved performance in the Morris water maze. These findings suggest that TREM2 is integral to controlling the Aβ plaque load by initiating a microglial phagocytic response to the aberrant protein accumulation. Interestingly, this effect appears to depend on the state of disease progression. APP/PS1-TYROBP^−/−^ mice display a reduction in the prevalence of Aβ oligomers and microglial activation compared to APP/PS1-TYROBP^+/+^ mice, indicating that a reduction in TREM2 signaling leads to beneficial effects in an AD mouse model [183]. Kim and colleagues recently found that TREM2 knockout was sufficient to cause the formation of endogenous murine Aβ plaques, and showed that TREM2 promotes the phagocytosis of Aβ by upregulating CD36 in microglia [184]. These data make it clear that TREM2 has a significant role in the clearance of abnormal protein deposits, although the mechanism by which this occurs has yet to be identified. TREM2 appears to be essential for the recognition of Aβ by microglia, but also counteracts the TLR-mediated inflammatory response [185], thereby having a pro-phagocytosis, but anti-inflammatory effect on microglia. In addition to the different ages and AD models used by various researchers, this may also account for the variations observed in the outcomes reported using TREM2-deficient AD mouse models.

More recently, tau models of AD were compared in the presence or absence of TREM2. Initially, knockout of TREM2 in the hTau model was shown to increase tau pathology, as well as increasing neurodegeneration and cognitive decline [186]. In contrast, a recent paper by Leyns and colleagues reported that neurodegeneration is reduced in the PS19-TREM2^−/−^ mouse model, compared to PS19-TREM2^+/+^ animals [187]. The authors found that there was reduced microgliosis and astrogliosis in PS19-TREM2^−/−^ mice, indicating that the reduced inflammatory activity may reduce neuronal damage. Although these results appear to be contradictory, it is worth noting that the PS19 model of tau pathology is more severe and the microglia may therefore already be in an overactive state. Reducing microglial phagocytosis in PS19-TREM2^−/−^ mice may lead to a reduction in neuronal damage, while causing an increase in inflammation in mice with less severe pathology via TLR activation, as observed in mouse models of Aβ with TREM2 deficiency. Microglial-neuronal co-culture has also recently been used to identify the effects of TREM2 during tau pathology, revealing that the tau pathology in neurons is reduced in the presence of microglial TREM2 [188]. Jiang and colleagues suggest that this is due to suppression of the microglial inflammatory response by TREM2, as its presence leads to a reduction in the transcription of inflammatory mediators. At this stage, the effects of TREM2 on tau pathology are still becoming clear and may differ depending on the pathology and age of the mouse model used. Although it seems that microglia expressing TREM2 are important for the removal of pathological tau species, it is also possible that over-activation of these cells, and the resultant chronic inflammation, have a detrimental effect on tau function.

### The CD33 SNPs confer differing risks for AD

CD33, also known as Siglec-3, is a type-I transmembrane protein from the sialic-acid-binding immunoglobulin- like lectin family, which is known to mediate intercellular communication and downregulate immune cell function. CD33 expression is also present on cells with phagocytic activity, such as macrophages, monocytes, dendritic cells, and microglia, and acts as an inhibitory mediator [189]. Following CD33 activation on human monocytes, PI3 kinase- and p38-MAPK-mediated inhibitory signal transduction has been reported to impair inflammatory-cytokine synthesis, leading to decreased levels of TNF-α, IL-8, and IL-1β [190]. More recently, downregulation of CD33 has also been associated with increased production of TNF-α [191].

CD33 was initially implicated in AD pathogenesis by GWAS in four well-characterized samples of AD families, where several risk single nucleotide polymorphisms (SNPs) were identified, with the highest risk being associated with rs3826656 [192]. GWAS has also shown that increased expression of CD33 is associated with cognitive decline in dementia patients and that SNP rs3865444 is upregulated in AD patient brains [193]. An increase in the expression of rs3865444 has also been reported to positively correlate with Aβ pathology upon patient autopsy [194]. Interestingly, a minor allele of rs3865444 (A) was found to be AD-protective [195], compared to the significant AD risk conferred by allele rs3865444 (C) [196]. Research into the mechanism behind these differences revealed that each allele altered the splicing of rs3865444 SNP mRNA, leading to increased expression of the rs3865444 (C) allele and thus increased CD33 expression [197], whereas the rs3865444 (A) allele led to translation of an inactive isoform of CD33 [198, 199]. One of these studies [198] also described the effects of CD33-knockout in the APP/PS1 mouse model, in demonstrating that APP/PS1-CD33^−/−^ mice displayed a significant decrease in amyloid plaque burden, without altering APP processing, compared to APP/PS1-CD33^+/+^ controls. CD33 was also shown to be primarily expressed by microglia, suggesting that it prevents the clearance of Aβ, thereby contributing to the risk of AD via failure of microglial phagocytic activity.

Interestingly, there appears to be a relationship between the effects of CD33 and TREM2 during AD. The AD-risk CD33 rs3865444 (C) allele is associated with increased TREM2 surface expression in monocytes, compared to rs3865444 (A), whereas blocking CD33 signaling leads to a reduction in TREM2 surface expression [200]. Measuring the levels of TREM2 and CD33 expressed by microglia at different time points following Aβ treatment revealed that TREM2 is expressed at a higher level initially, correlating with increased microglial phagocytosis [201]. Later time points in these experiments revealed that TREM2 expression subsequently decreases, whereas CD33 expression increases in association with diminished phagocytic capabilities. In addition, CD33 was shown to be downregulated in APP/PS1-TYROBP^−/−^ mice, which had reduced microglial reactivity to Aβ and performed better in spatial learning tests [183]. Overall, this evidence suggests that the receptors CD33 and TREM2 may work as an on-off switch for microglial phagocytosis in healthy individuals, a process which is altered during AD where gene expression is skewed towards heightened microglial reactivity and a pro-inflammatory response.

## The metabolic disorders and AD

Aging is also associated with other chronic disorders, including metabolic syndromes such as diabetes, obesity, and hypertension, which also appear to contribute to overall inflammation and the exacerbation of AD. Thus, in many cases, it is difficult to study the contribution of aging to AD in isolation from other age-dependent disorders. Type 2 diabetes mellitus (T2DM), for instance, has been shown to increase the risk of developing AD, as well as contributing to the worsening of neurological symptoms [202]. Epidemiological studies have reported that insulin resistance and T2DM elevate the risk of mild cognitive impairment progressing to AD [203, 204]. Failure of the insulin receptor in the synaptosomes of a diabetic mouse model (db/db) leads to accelerated Aβ aggregation in vitro [205], and similarly, excess peripheral insulin has been shown to increase hippocampal Aβ accumulation in APP/PS1 mice, an effect which is exacerbated by age [206]. Overall, obesity and T2DM exacerbate Aβ and tau pathologies, accelerating cognitive decline and worsening neuroinflammation in both middle age and old mice. Another interesting link between insulin resistance and Aβ accumulation is IDE, which is responsible for not only the degradation of excess insulin, but also the degradation of Aβ, as discussed earlier. Farris and colleagues have suggested that excessive insulin circulation during insulin resistance may lead to reduced degradation of Aβ [207]. Insulin resistance has also been found to alter tau dephosphorylation via inhibition of adenosine monophosphate-activated kinase [208], and tau hyperphosphorylation has been observed in the hippocampi of the fa/fa rat model of obesity, with the extent of phosphorylation being increased in aged rats [209]. Interestingly, the relationship between T2DM and AD does not appear to be one sided, with intrahippocampal injection of Aβ leading to decreased insulin sensitivity in male rats [210]. The ApoE4 genotype has also been implicated in the effects of obesity on AD, whereby a high-fat diet increased Aβ pathology in the familial AD (FAD) mouse model expressing ApoE4 but not ApoE3 [211], and was shown to worsen the cognitive decline associated with insulin resistance [212].

Although primarily associated with metabolism, obesity, hypertension, and T2DM have also been shown to lead to increased peripheral inflammation. Adipose tissue has been reported to produce pro-inflammatory cytokines [213], particularly TNF-α, which can impair insulin signaling, thereby increasing the risk of insulin resistance [214]. The excessive release of pro-inflammatory mediators, including TNF-α and IL-6, is likely due to the accumulation of macrophages in the adipose tissue of obese individuals [215]. Pro-inflammatory cytokine release has also been associated with a high-fat diet [216]. In patients with both T2DM and hypertension, the plasma levels of IL-6 were significantly increased [217], suggesting an inflammatory link within disorders classified as metabolic syndrome. Adipose tissue inflammation occurs prior to hepatic tissue inflammation in insulin-resistant, obese C57BL/6 mice, likely indicating that it contributes to the development of insulin resistance [218], with similar results being reported in CD-1 mice [219]. Further, in human patients with T2DM, the NLRP3 inflammasome is activated, leading to the upregulation of IL-1β and IL-18 [220], with a receptor agonist of the latter currently being tested in clinical trials for the treatment of T2DM [221]. Likewise, the NLRP3 inflammasome is also activated during hypertension [222]. Interestingly, the gene expression profiles of cultured peripheral immune cells have been shown to change in response to high glucose conditions and T2DM, affecting neutrophil movement [223], increasing monocyte activation markers [224] and increasing monocytic TLR expression [225]. Monocytes and neutrophils from T2DM patients have also been found to have increased pro-inflammatory cytokine and TLR expression, with the extent of this effect changing based on glycemic control [226]. Inflammatory mediators also have a causative effect in insulin resistance, with TNF-α being found to increase phosphorylation of insulin receptor substrate-1, which leads to diminished insulin signaling [227]. In addition, IL-1β, IL-6, and many other pro-inflammatory mediators have been implicated in defective insulin signaling, leading to insulin resistance and eventually T2DM [228–230]. Interestingly, Dhande and colleagues found that hypertension-protective stimulation of angiotensin receptor AT2 leads to anti-inflammatory IL-10 release, although the mechanism through which this occurs has yet to be determined [231].

In the brain, research has demonstrated that T2DM, a high-fat diet, and obesity cause cognitive decline and impairment, in both mouse models and human patients [232–235]. Hypertension has been shown to cause synaptic loss in wild-type mice [236], as has obesity in a rat model [237]. The same study also found that obesity caused phenotypic changes in the microglia of the rat prefrontal cortex, and led to cognitive decline [237]. Similar results have been reported in mice, where obesity activated microglia, causing excessive synaptic pruning and loss [238]. Overall, these data suggest that excessive inflammatory mediators produced by adipose tissue during metabolic syndrome affect the brain, stimulating microglia and causing synaptic pruning, and perhaps initiating the accumulation of Aβ. Although mechanistic studies are still necessary to directly link the changes in the immune system caused by metabolic disorders and AD, these findings reinforce the complexity of the relationship between inflammation and genetic and lifestyle risks, which may act cumulatively to increase the risk of developing AD.

## Acute damage to the brain triggers inflammation and increases AD risk

Epidemiological data point to TBI as a major risk factor for AD, although a direct link with AD has been inconsistently reported [239]. A recent analysis of the literature found that the relationship between TBI and dementia in human patients is likely dependent on TBI severity and frequency, which may account for varying reports of correlation [240]. In humans, TBI was shown to cause increased production of soluble Aβ in resected cortical tissue, and it was also reported that the ApoE genotype of the patient may alter their risk for AD in later life [241]. Further, in approximately 30% of fatal TBI cases, Aβ deposits were identified in the neocortex [242]. Johnson and colleagues also found Aβ deposits at a greater density in the post-mortem brains of TBI patients than in non-injured controls, and demonstrated that tau NFTs were present in approximately one third of TBI patients’ tissue [243]. A controlled cortical impactor was used to test this in a rat model, revealing increased activation of inflammatory cells, increased expression of APP and increased tau phosphorylation [244]. Conversely, it has also been proposed that the increase in APP following acute TBI is beneficial, with APP^−/−^ mice having worse behavioral outcomes than APP^+/+^ controls [245], possibly due to the neuroprotective peptide secreted APPα [246, 247]. Although the upregulation of APP may be important for neuronal survival, it could also lead to the production of Aβ in some cases of TBI. Overall, it appears that TBI worsens Aβ and tau pathology, via increased neuroinflammation.

TBI generally involves both a primary and a secondary injury; the primary injury is caused by a mechanical force during the acquisition of the injury, while the secondary injury usually follows the disruption of the BBB, leading to brain swelling/edema, increased inflammation and hyperexcitability. Patient complications generally arise from the secondary injury, as a result of excessive neuronal loss. Microglia have been shown to activate within minutes of TBI in mouse models, resulting in positive survival outcomes in mice compared to those that could not mount a microglial response [248]. Activation of microglia stimulates the release of pro-inflammatory cytokines and chemokines, as well as danger-associated molecular pattern (DAMP) molecules, which signal cell death and further stimulate phagocytic microglia [249]. Damaged neurons have also been found to release pro-inflammatory signals during the primary injury [250]. During the secondary injury, increased levels of pro-inflammatory mediators (e.g., IL-1β, IL-6, IL-8, TNF-α) are associated with a poorer prognosis, increased intracranial pressure, and mortality in human patients [251]. In a rat model of repetitive mild TBI, IL-6 and TNF-α were also expressed at higher levels than in rats modeling single mild TBI, indicating that repetitive injuries can cause chronic inflammation [252]. Interestingly, in rats where the more severe Feeney model of TBI was used, researchers found that, although IL-1β expression was initially upregulated following injury, IL-18 was chronically expressed [253]. This may indicate that IL-18 has a prolonged effect on TBI, perhaps related to the secondary injury and ongoing recruitment of peripheral inflammatory cells.

In addition to microglia, astrocytes are also responsible for regulating the inflammation associated with TBI and can modulate BBB permeability during injury. Astrocytes also express TLRs and are therefore able to react to inflammatory stimuli, leading to the release of pro-inflammatory mediators [254]. The activation of astrocytes can cause astrocyte swelling/edema, which poses a significant risk to the recovery of TBI patients following injury, and is regulated by the activation of astrocytic NF-κB [255]. Despite this, reactive astrocytes are also integral to endothelial repair of the BBB following [256]. Astrocytes have long been associated with BBB health, with the interaction between astrocytes and endothelial cells being integral to its correct function, including its permeability to the influx of peripheral immune cells following TBI [33, 257]. The increase in the pro-inflammatory cytokines TNF-α and IL-1β in the hours following TBI may be responsible for the increased permeability of the BBB, initially in order to recruit immune cells. TNF-α has been shown to increase the permeability of endothelial monolayer cultures via actin remodeling in vitro [258, 259], and transgenic overexpression of IL-1β in mice was found to increase BBB permeability [260]. In addition, increased expression of chemokines and chemokine receptors following TBI, including CXCL2, CXCR2, CCL2, CXCR4, and chemokine-like factor 1 (CKLF1), has been shown to further permeabilize the BBB [261–264] and attract neutrophils [265, 266]. This causes a significant influx of peripheral immune cells into the brain following TBI, which has been found to worsen edema and neuronal damage [267–269].

Together, these studies suggest that the timing of pro-inflammatory signaling may be the key to survival following injury or illness. The initial pro-inflammatory event is necessary to allow immune cells to clear infection or debris, but if prolonged, it can cause excessive damage and secondary diseases including AD. Although it is not entirely clear how TBI might initiate AD, it is worth noting that the ApoE4 allele is associated with both AD risk and increased severity of TBI [270], and may therefore also play a significant role in the risk that TBI poses in relation to AD. Interestingly, a link was also recently made between ApoE4, TBI, and tau phosphorylation. Cao and colleagues found that ApoE4 knock-in mice had significantly more phosphorylated tau following blast TBI than ApoE3 knock-in mice, via activation of GSK3β [271]. In addition, recent transcriptional profiling of ApoE4 and ApoE3 knock-in mice at 14 days post-TBI found significant upregulation in TREM2, TYROBP, C-type lectin domain containing 7A, Cd68, and CX3CR1 compared to the sham control [272]. Not only are these proteins related to phagocytosis, but they have also been investigated thoroughly with respect to AD, where malfunction in TREM2 and Tyrobp proteins leads to increased AD risk. Out of 11 epidemiological reports published between 2005 and 2015, only two accounted for ApoE genotype [240], with one finding an additive effect of ApoE4 genotype and head injury in the risk of developing dementia [273], and the other reporting little evidence for a relationship between ApoE4 and dementia [274]. It is possible that AD is triggered by TBI in individuals who already carry an AD risk gene, which may explain conflicting literature if the genetic risks are not considered and also suggests that genetic and environmental risk factors could act cumulatively.

## The microbiota, inflammation, and AD

Interest in the role that the gut microbiota plays in disease has increased in recent years, as evidence of its importance in maintaining the normal physiology and health of the host has grown. Bacteria, fungi, archaea, virus, and protozoa collectively form our gut microbiota, acting in a symbiotic manner beneficial to the host. Changes in the composition of this complex ecosystem, referred as dysbiosis, has been associated with aging and the development of inflammatory and CNS disorders, including AD [275]. An infectious origin of AD has long been postulated, with ethiological hypotheses suggesting chronic that infection with various bacteria, viruses, parasites, and fungi may contribute to AD. However it was only recently that researchers started to explore the role of the gut microbiome in the pathogenesis of AD.

Aging causes the degeneration of enteric nervous system, alterations in gastrointestinal motility, and perturbations in small intestinal permeability and the mucosal defense system. This degeneration may promote the development of gastrointestinal disease, affect the local and systemic inflammatory status, and deeply influence both the composition and function of the resident microbiota [276], reducing its diversity and stability [277, 278]. These changes lead to a progressive decline in immune function associated with a chronic pro-inflammatory response [279, 280]. As previously discussed, immunosenescence also has severe consequences in the brain, such as BBB breakdown, and microglial hyper-activation and eventual senescence, which contribute to the development of AD [281]. Age-related changes in the gut microbiota have been shown to impact behavioral and cognitive functions in mice and support the relevance of the alteration in gut permeability and peripheral inflammation in mediating these effects [280]. An aged gut microbiota has also been shown to promote systemic immunosenescence when transferred to germ-free mice [282]. This suggests that the gut microbiota dysbiosis observed in the elderly could contribute to peripheral inflammation, therefore exacerbating neuroinflammation and AD.

In addition to aging, there are many other factors that can affect gut microbiota populations. Changes in gut the microbiota can trigger an immune system response and the microbiome of patients with inflammatory diseases. For example, in multiple sclerosis patients, researchers found that specific species of gut bacteria were upregulated or downregulated and that microbiota transplants from patients to germ-free mice led to an autoimmune response, which was not observed with microbiota transplants from healthy controls [283]. Due to this tight cross-talk between the gut microbiota and the immune system, a wide variety of external factors which affect the former can ultimately affect systemic inflammation and promote disease progression. Thus, it is not surprising that most of the factors described as risks for AD also influence the gut microbiota, including obesity [284], diet [278], T2DM [285, 286], chronic stress [287, 288], and the use of antibiotics [289]. Interestingly, as the gut microbiota can be modified by diet, many of these effects were effectively restored and even prevented by treatment with probiotics [290, 291].

A recent study which compared the composition of the gut microbiome in participants with and without AD revealed that the gut microbiome of AD participants had decreased microbial richness and diversity [292]. This study reported that AD patients have a low abundance of *Firmicutes* and *Bifidobacterium* and a characteristic increase in the abundance of *Bacteroidetes* [292]. This difference in microbial diversity correlated with cerebrospinal fluid biomarkers of AD pathology [292]. Gut bacterial taxa in cognitively impaired elderly participants were studied, revealing that the abundance of the pro-inflammatory *Escherichia/Shigella* was increased, whereas anti-inflammatory *E. rectale* was reduced, which correlated with cognitive impairment and brain amyloidosis [293]. Studies of AD mouse models and germ-free mice supported the latter findings. The APP/PS1 mouse model showed a distinct microbial signature, with an increase in *Rikenellaceae* and decreased *Allobaculum* and *Akkermansia* [294]. Interestingly, reduced levels of *Akkermansia* in the gut microbiota is also associated with mouse models of obesity and T2DM [295], two potentially modifiable risk factors for AD. Researchers also found that young and old APP/PS1 mice raised under germ-free conditions had reduced cerebral Aβ pathology when compared with control mice, together with reduced microgliosis [295]. Increased cerebral Aβ pathology was observed following a microbiota transplant from conventionally raised APP/PS1 mice, whereas transplant from non-transgenic mice did not increase Aβ levels to the same extent [295]. Germ-free-APP/PS1 mice exhibited increased expression of the Aβ-degrading enzymes IDE and neprilysin degrading enzyme compared to conventionally raised APP/PS1 mice, suggesting that the gut microbiota influences the clearance of Aβ via degradation [294]. Data supporting this finding were obtained in APP/PS1 mice following life-long antibiotic treatment, which induced dysbiosis [296]. Interestingly, elevated levels of APP were found not only in the brain but also in the different gut districts from the 5xFAD mouse model, and these changes were associated with a distinct fecal microbiota profile, indicating an increase in pro-inflammatory species (e.g., *Clostridium leptum*) [297]. Hence, these studies provide evidence that the diversity of gut microbiota can modulate host innate immunity mechanisms that impact amyloidosis, thereby affecting the progression of AD. The imbalances in gut microbiota composition together with the increased BBB permeability that occurs with age may lead to the translocation of microbes or microbial components from the gut to induce neuroinflammation [298]. In this regard, coincubation of Aβ with LPS has been shown to potentiate Aβ fibril formation [299], and systemic administration of LPS in non-transgenic and transgenic AD mice induces neuroinflammation, Aβ deposition, and tau pathology [115, 128, 300, 301]. In addition, the presence of bacterial LPS has been reported in brain lysates from the hippocampus and superior temporal lobe neocortex of AD brains [302]. In a similar study, the levels of LPS and gram-negative *E. coli* fragments were greater in the post-mortem brain parenchyma and blood vessels of patients with AD compared with controls, and colocalized with amyloid plaques [301]. These studies provide evidence that implicates gut microbiome-derived LPS as an important internal contributor to inflammatory degeneration in the CNS.

Recent studies have demonstrated that manipulating the gut microbiota can influence host innate immunity mechanisms which impact cerebral amyloid deposition and neuronal plasticity processes [294, 296, 303]. Promising preclinical data suggest that restoring homeostasis in the host-microbiota interactions in the elderly could be a way to improve intestinal inflammation and function, and influence their systemic inflammatory status. Prebiotics (nutritional support for bacteria), probiotics (live bacteria), symbiotics (combination of probiotics and prebiotics), certain antibiotics, and pharmacological inhibitors or activators have been shown to modulate the microbiota and correct inflammatory conditions in the elderly, thereby providing a potential means to counteract the development or progression of neurodegenerative diseases [304]. Despite the potential to slow or reverse pathology by modifying the diversity of the gut microbiota, further research is needed to better understand its complex interaction with AD biology, which could be essential to take preventive measure such as early diagnosis.

## Lifestyle as an anti-inflammatory modifier of AD risk

Learning and memory improvements have been observed when lifestyle factors are changed (i.e., increased exercise and low-fat diet). Considering the vast and varied ways in which AD and inflammation appear to intersect, it is fitting that researchers have also investigated whether inflammation could be modified to reduce the risk of AD. In the APP/PS1 mouse model of AD, changing from a high-fat diet to a low-fat diet led to a return to baseline levels of neuroinflammation and reduced the levels of insoluble Aβ [305]. A study focused on improving the nutrient status of APP/PS1 mice found that an improved diet also led to better performance in the Morris water maze [306]. Moreover, physical exercise has also been shown to rescue behavioral deficits caused by inflammation. In rats with induced neuroinflammation, access to a treadmill or running wheel led to improved escape latency and distance traveled to find a submerged platform in the Morris water maze [307]. As the induction of LTP is inhibited during neuroinflammation, researchers determined that exercise improved behavioral deficits by rescuing LTP. Aged mice have increased baseline neuroinflammation, expressing IL-1β and IL-10 at a greater level in the hippocampus than adult mice [308]. This effect can be attenuated by access to a running wheel, decreasing hippocampal cytokine levels as well as reducing the depressive “sickness behavior” observed in aged mice [308]. The behavioral deficits caused by intracerebroventricular injection of Aβ were also shown to be attenuated when Swiss-albino mice performed swimming training [309]. This exercise training also reduced the levels of the pro-inflammatory cytokines IL-6 and IL-4 in both the prefrontal cortex and hippocampus via a decrease in NF-κB activity [309]. In the 3xTg-AD mouse model, 8-week voluntary running wheel exercise led to reduced neurodegeneration and apoptosis, as well as a reduction in the pro-inflammatory cytokines TNF-α and IL-6, compared to 3xTg-AD mice which did not [310]. Similar results were observed in 3xTg-AD mice which were administered insulin sensitizing drugs [311]. Kang and colleagues also showed that exercise could significantly reduce Aβ plaque load in PS2 transgenic mice, simultaneously reducing TNF-α levels and apoptosis [312]. Given that inflammation can trigger Aβ synthesis, and that Aβ can trigger inflammation, it is difficult to determine how the effects of exercise occur, particularly as there is also evidence that exercise reduces BACE1 and other facilitators of the Aβ production pathway [313]. However, recent research from Lu and colleagues using a rat model of AD showed that the positive effects of exercise were due to a microglial shift from the pro- to the anti-inflammatory phenotype, thereby reducing neuronal death [314]. This suggests that there is a direct, positive effect of exercise on the response of microglia, which may be able to reduce their aberrant effects during AD.

The effects of exercise have seldom been investigated with respect to tau pathology, although the available data appear promising. Providing THY-Tau22 mice with access to a running wheel for 9 months resulted in reduced tau phosphorylation and a significant decrease in TNF-α and IL-1β [315]. However, short-term exercise was shown to increase microglial reactivity and the release of pro-inflammatory cytokines in both non-transgenic and Tg601 mice, together with an increase in phosphorylated tau in the transgenic mice [316]. This may be due to the shorter length of the exercise period, such that the positive effects observed with running, including brain-derived neurotrophic factor release, have not yet occurred. Our understanding of the effects of exercise on tau is still to reach consensus with respect to the involvement of inflammation; however, these data point towards a dose-dependent effect of exercise in animal models.

The effects of exercise and improved diet on cognition are clear, and these lifestyle changes have been tested on human patients with promising results. A meta-analysis of research in humans has shown that there is a distinct positive correlation with weight loss and improvement of cognitive functions [317]. Strength training in older women was found to improve their cognitive ability, while preventing the increase in TNF-α seen without such training [318]. In addition, high levels of pro-inflammatory IL-12p40 were found to correlate with rapid cognitive decline, an effect which was attenuated by exercise [319]. This suggests that exercise can modify neuroinflammation in a way that translates to improved cognition. However, further research into standardizing the optimal type and amount of exercise would be beneficial for the field.

## Conclusions

**Fig. 4:**
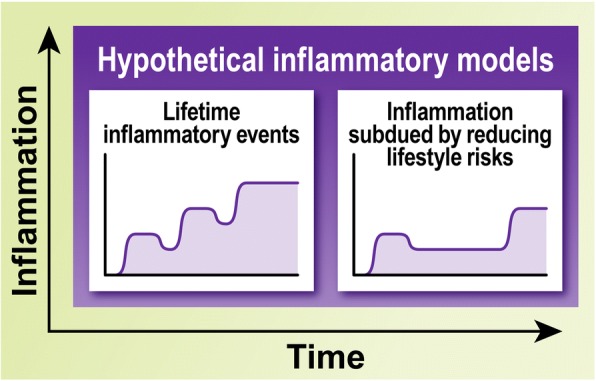
Proposed model of immunosenescence over time. We suggest that immunosenescence is related to the cumulative effects of many inflammatory events throughout life, including infection, metabolic disorders, or genetic risks. It may be possible to reduce immunosenescence by modifying lifestyle, leading to a reduced cumulative effect, which may subsequently decrease the risk of developing AD

Although the inflammatory response to acute incidents is tightly regulated, chronic inflammation is ineffective and can lead to worsened outcomes during many diseases. This is true in the case of AD, where inflammation becomes detrimental to neuronal survival and accelerates cognitive decline. The fact that the risks for AD involve various changes to homeostatic inflammatory responses is evidence of the clear clinical influence of inflammation on disease progression. A multitude of inflammatory events can occur throughout an individual’s lifetime, and microglial priming observed during aging suggests that the baseline level of inflammation can shift over time. This may lead to a cumulative AD risk caused by a lifetime of inflammatory conditions, some of which involve genetic and lifestyle factors (Fig. 4).

**Fig. 5:**
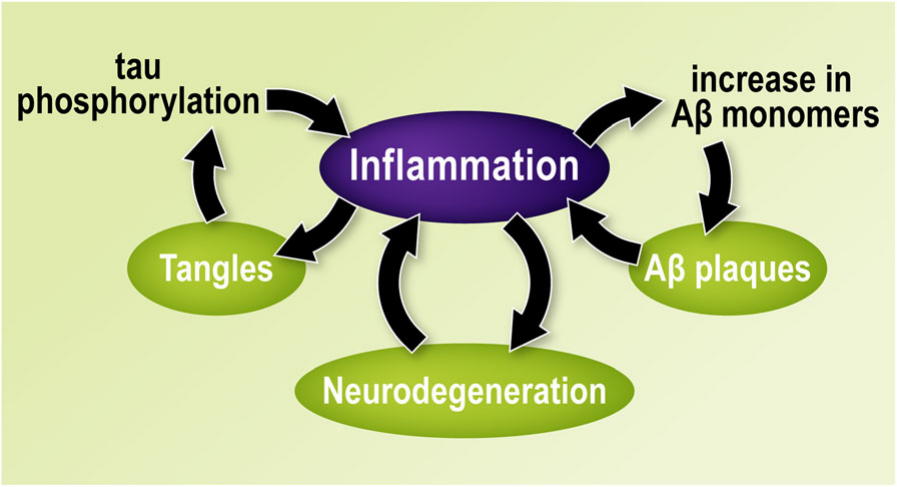
Inflammation uniquely affects each of the hallmarks of AD. Each hallmark of AD has a unique relationship with inflammation, whereby it can both be triggered by and trigger inflammation. Aβ plaques activate microglia and trigger their pro-inflammatory signaling pathways, which can cause increased Aβ production. Tau phosphorylation can be caused by the production of Aβ plaques, as well as by increased inflammatory signaling. Tau hyperphosphorylation and tangles can also lead to increased inflammation. Finally, neurodegeneration can be caused by an excessive microglial response, while neuronal debris from neurodegeneration can lead to further stimulation of microglia

Inflammation influences each of the hallmarks of AD (i.e., Aβ, tau and synaptic and neuronal loss), and to complicate this further, exerts different effects at different stages of AD progression (Fig. 5). For example, attenuating pro-inflammatory phagocytosis during the initial stages of Aβ plaque accumulation has detrimental effects, but if pro-inflammatory signaling becomes chronic, it causes tau pathology to worsen and increases synaptic loss. Chronic inflammation therefore presents an interesting dilemma: would it improve patient outcomes to be able to reactivate microglia, enabling them to continue to clear Aβ plaques and extending pro-inflammatory signaling, or would resolution of inflammation lead to better patient outcomes? Stimulation of phagocytosis without excessive pro-inflammatory signaling would be ideal in the treatment of AD. Clearly, the timing of pro- and anti-inflammatory effects is key to both the pathology of AD and potentially its treatment.

Extensive research is therefore needed to find a way in which to provide the balance between pro- and anti-inflammatory responses to resolve the inflammation with appropriate timing, thereby avoiding the risk factors associated with chronic inflammation. However, in order to achieve this, tools such as immuno-based biomarkers need to be developed to allow the early detection of inflammatory diseases, so as to be able to modulate the inflammatory response and delay the onset and/or the progression of the disease. While scientists focus their efforts on achieving this aim, it is also important to highlight the relevance of actively promoting good habits, such as regular exercise, eating a healthy diet and avoiding harmful lifestyle risks, to minimize inflammation and its contribution to AD risk.

## Abbreviations

ABC: ATP-binding cassette
AD: Alzheimer’s disease
ADAM: A disintegrin and metalloproteinase
AMPAR: Α-Amino-3hydorxy-5-methyl-4-isoxazolepropionic acid receptor
AP-1: Activator protein 1
ApoE: Apolipoprotein E
APP: Amyloid precursor protein
Aβ: β-Amyloid
BACE1: Beta-secretase-1
BBB: Blood-brain barrier
C1q: Complement component 1q
CCL: CC-chemokine ligand
CD: Cluster of differentiation
CDK5: Cyclin-dependent kinase 5
CKLF1: Chemokine-like factor 1
CNS: Central nervous system
COX-2: Cyclooxygenase-2
CX3CR1: CX3C chemokine receptor 1
CXCL: CXC-chemokine ligand
DAMP: Danger-associated molecular pattern
EP4: Prostaglandin E2 receptor 4
ERK: Extracellular signal-regulated kinase
FAD: Familial AD
GSK3β: Glycogen synthase kinase-3β
GULP1: Engulfment adapter PTB domain containing 1
GWAS: Genome-wide association studies
Iba1: Ionized calcium binding adaptor molecule 1
IDE: Insulin degrading enzyme
IFN-γ: Interferon-γ
IL: Interleukin
iNOS: Inducible nitric oxide synthase
JNK: C-Jun N-terminal kinase
LOX: Lipoxygenase
LPS: Lipopolysaccharide
LRP-1: Lipoprotein receptor-related protein-1
LTP: Long-term potentiation
LXA_4_: Lipoxin A_4_
MEGF10: Multiple epidermal growth factor-like domains 10
MerTK: C-MER proto-oncogene tyrosine kinase
MHC-II: Major histocompatibility complex-II
MLA: Monophosphoryl lipid A
MMP: Matrix metalloproteinase
NALP3: NACHT, LRR and PYD domains-containing protein 3
NFTs: Neurofibrillary tangles
NF-κB: Nuclear factor-κB
NLRP3: Nlr family pyrin domain containing 3
NMDAR: *N*-methyl-d-aspartate receptor
p38-MAPK: p38 mitogen-activated protein kinase
PGE_2_: Prostaglandin E_2_
PHF-1: PHD finger protein-1
PPAR-γ: Peroxisome proliferator-activated receptor-γ
RAGE: Receptor for advanced glycation end products
RvE1: Resolvin E1
SNPs: Single nucleotide polymorphisms
SRA: Scavenger receptor A
STAT3: Signal transducer and activator of transcription 3
sTREM: Soluble TREM2
T2DM: Type 2 diabetes mellitus
TBI: Traumatic brain injury
TGF-β: Transforming growth factor-β
TLR: Toll-like receptor
TNFR1: TNF-type 1 death receptor
TNF-α: Tumor necrosis factor-α
TREM2: Triggering Receptor Expressed on Myeloid Cells 2
TYROBP: TYRO protein tyrosine kinase binding protein
